# Lasting Increases in Neuronal Activity and Serotonergic Receptor Expression Following Gestational Chlorpyrifos Exposure

**DOI:** 10.1523/ENEURO.0195-25.2025

**Published:** 2026-01-02

**Authors:** Jeffrey A. Koenig, Nathan Cramer, Kara Kessler, Jimmy Olusakin, Mary Kay Lobo, Asaf Keller

**Affiliations:** Department of Neurobiology and UM-MIND, University of Maryland School of Medicine, Baltimore, Maryland 21201

**Keywords:** neurotoxicity, organophosphate, parvalbumin, pesticide, serotonin, somatosensory

## Abstract

Perinatal exposure to the organophosphorus insecticide chlorpyrifos (CPF) is associated with an increased incidence of neurodevelopmental disorders, such as autism spectrum disorder. While these behavioral detriments have been modeled in rodents, the underlying functional alterations in the developing brain are largely unknown. Previous reports using a rat model have identified alterations to both inhibitory synaptic transmission and serotonergic (5-HT) receptor binding in the cortex following developmental CPF exposure. Here, we use a rat model of gestational CPF exposure to investigate whether this altered inhibitory activity is driven by increased spontaneous firing of inhibitory interneurons and altered 5-HT receptor expression. Using cell-attached ex vivo electrophysiology in young rats of both sexes, we identified a significant increase in the number of spontaneously firing neurons in the somatosensory cortex of CPF-exposed offspring. Analysis of action potential metrics identified a subset of these neurons as fast-spiking parvalbumin (PV) interneurons. Immunohistochemical labeling of c-Fos, a marker of neuronal activity, further revealed a pronounced increase in activity of neurons of the somatosensory cortex in both juvenile and adult rats that had been gestationally exposed to CPF. Finally, RNAscope in situ hybridization showed an increase in the expression of the inhibitory receptor 5-HT_1B_ in PV neurons of male offspring. The preliminary data reported here suggest that gestational exposure to CPF may result in persistent hyperexcitation of the somatosensory cortex. These neurophysiological effects may contribute to the established behavioral outcomes resulting from gestational exposure to CPF and offer guidance for novel preventative interventions.

## Significance Statement

We report persistent increases in spontaneous neuronal firing in the somatosensory cortex following a brief gestational exposure to the organophosphorus insecticide chlorpyrifos (CPF) in rats. This occurred in conjunction with increased expression of the 5-HT_1B_ receptor measured specifically in PV interneurons. The hyperexcitability of the somatosensory cortex described here agrees with the established hypersensitivity to sensory stimulation seen in neurodevelopmental disorders such as autism spectrum disorder. These preliminary results offer a possible mechanistic framework underlying the neurophysiological effects associated with early life exposures to the organophosphorus insecticide CPF.

## Introduction

Chlorpyrifos (CPF) is an organophosphorus insecticide ubiquitously used in agriculture. Like other organophosphorus compounds, CPF is toxic to nontarget species, including humans, primarily through potent inhibition of the enzyme acetylcholinesterase (Taylor, 2011). The acute cholinergic effects of CPF exposure have long been understood, but more recent attention has been given to subacute environmental exposures that are not associated with overt signs and symptoms of acute toxicity or significant inhibition of acetylcholinesterase. Several longitudinal studies have linked these exposures, especially during pregnancy, to an increased incidence of neurodevelopmental disorders such as attention deficit/hyperactivity disorder and autism spectrum disorder (Rauh et al., 2012; Shelton et al., 2014; Schmidt et al., 2017). While this has led to more restrictions on the application of CPF, its worldwide use continues to grow (ECHA, 2023).

The developmental neurobehavioral effects of CPF have been modeled in several animal species, including rats, mice, and guinea pigs. Perinatal exposures of these animals to doses of CPF that do not induce acute toxicity results in increased anxiety-related behaviors, learning and working memory deficits, and alterations to somatosensory function (Levin et al., 2001; Aldridge et al., 2005; Muller et al., 2014; Mamczarz et al., 2016). Investigations into noncholinergic neurochemical alterations have primarily implicated serotonergic (5-HT) signaling systems. Following perinatal CPF exposure in rats, binding of 5-HT_1A_ and 5-HT_2_ receptors is increased at least until 5 months of age (Aldridge et al., 2003, 2004; Slotkin and Seidler, 2005). However, 5-HT receptors are diversely expressed, both pre- and postsynaptically, and on both inhibitory and excitatory neurons (Celada et al., 2013; Palacios, 2016), making it difficult to predict the functional consequences of this increase in receptor binding. Furthermore, whether these changes in receptor binding reflect changes in receptor expression or in receptor affinity remains unknown.

Following subacute gestational exposure to CPF in rats, there are persistent alterations to inhibitory synaptic signaling and use-dependent plasticity in the rat somatosensory (barrel) cortex (Koenig et al., 2025). We hypothesize that these anomalies are driven by disinhibition and increased activity of parvalbumin (PV) neurons. PV neurons are a unique class of fast-spiking inhibitory neurons that provide dense input and inhibition to neighboring cells (Packer and Yuste, 2011). In addition to serving as a primary inhibitory input to pyramidal neurons and to other PV neurons, they are critical for the proper refinement of use-dependent plasticity (Trachtenberg, 2015; Vickers et al., 2018; Rupert and Shea, 2022). Dysregulation of PV neuron activity has been implicated in several neurological disorders, including autism spectrum disorder (Marín, 2012; Filice et al., 2020). PV neurons are particularly sensitive to toxin exposure during development (Dendrinos et al., 2011; Stansfield et al., 2015; Reid et al., 2021).

In the present study, we test the hypothesis that changes in cortical functions after gestational exposure to CPF result in lasting in vivo increases in neuronal activity and in cell-specific changes in 5-HT receptor expression. We focus on the somatosensory (barrel) cortex, as its development is highly characterized (Erzurumlu and Gaspar, 2012; Yang et al., 2018) and because both PV neurons and proper 5-HT signaling play a key role in its maturation and function (Nowicka et al., 2009; Miceli et al., 2013, 2017; Sachidhanandam et al., 2016; Teissier et al., 2017; Kimura and Itami, 2019).

## Materials and Methods

### Animals and treatment

All procedures adhered to the Guide for the Care and Use of Laboratory Animals and approved by the Institutional Animal Care and Use Committee at the University of Maryland School of Medicine. Male and female Long–Evans rats were acquired from Charles River Laboratories and bred in our vivarium. Animals were housed under a standard 12/12 h light/dark cycle (0600 on/1800 off) and had *ad libitum* access to food and water. Pregnancy was confirmed by the presence of sperm in a vaginal lavage collection and set as Gestational Day (GD) 0. CPF (Chem Service) was dissolved in a 50/50 mixture of DMSO/peanut oil and injected subcutaneously (0.5 ml/kg) once daily on GD 18–21 at a dose of 5 mg/kg. Previous studies have routinely used DMSO (1.0 ml/kg) as the vehicle for CPF administration in rat dams (Levin et al., 2001; Garcia et al., 2003; Aldridge et al., 2005). The dose of DMSO administered here (0.25 ml/kg) was reduced to limit any potential off-target toxicity (Galvao et al., 2014). The use of an identical dose of DMSO in control animals further mitigates this concern. In addition, the dose of DMSO administered in the current study (0.25 ml/kg) is well below the highest no-observable-adverse-effect level previously reported in rats (Gad et al., 2006). Control dams received vehicle (DMSO/peanut oil) injections on the same schedule. Litters were culled to a maximum size of 10 by PND 3 through random pup selection. Dams were pair-housed until GD 18. Offspring not collected as juveniles (PND 12–20) were weaned at PND 21 and pair-housed by sex until tissue collection at the adult time point (>PND 90). Both male and female progeny were used in all experiments, unless noted otherwise in Results.

The CPF exposure dose (5 mg/kg) was selected to probe mechanisms driving the persistent neurodevelopmental alterations associated with subacute, nonsymptomatic, CPF exposure. A similar dose was used in a series of studies by Slotkin and collaborators (Aldridge et al., 2003, 2004; Slotkin and Seidler, 2007). This exposure paradigm significantly inhibits brain AChE in offspring of treated dams. However, AChE enzymatic activity returns to control levels by PND 12 (Koenig et al., 2025), a period preceding the endpoints presented here. We recognize that this dose does not necessarily reflect levels of environmental exposure in the human population.

### Slice electrophysiology

As previously described (Alipio et al., 2021), animals (PND 12–20) were deeply anesthetized with ketamine/xylazine, the brains were removed, and 300 μm coronal slices containing the primary somatosensory cortex were prepared. Slices were placed in a recording chamber continuously perfused (1.5 ml/min) with carbogen saturated artificial cerebrospinal fluid (ACSF) containing the following (in mM): 124 sodium chloride, 2.5 potassium chloride, 1.25 monosodium phosphate, 24 sodium bicarbonate, 12.5 glucose, 2 magnesium sulfate heptahydrate, and 2 calcium chloride dihydrate (Sigma-Aldrich). The recording pipette (4–6 MΩ) was filled with ACSF solution. Inhibitory interneurons in the superficial layers (L 2/3 and L 4) of the barrel cortex were targeted based on morphology and lack of prominent apical dendrite. Cell-attached recordings were performed in voltage clamp mode (0 pA holding current) with a loose seal resistance (<200 MΩ; Perkins, 2006). Seal resistance was measured before and after recording to ensure patch stability. A cell was considered “active” if there were any spontaneous fast-current transients representing action potentials within an ∼3 min recording period.

### Histology

c-Fos expression was visualized through immunohistochemistry. Both juvenile (PND 12–13) and adult (>PND 90) animals were used in this experiment. Juvenile animals were removed from their littermates and dam before being quickly (<15 min) killed. Adult animals were monitored in their home cage, absent enrichments, for 2 h prior to collection, as previously described (Filipkowski et al., 2000). There was no discernible, qualitative difference in the mobility or other activity between animals from CPF- and vehicle-exposed groups. Animals were deeply anesthetized by intraperitoneal injection of ketamine/xylazine (80 and 10 mg/kg) and transcardially perfused with ice-cold PBS followed by 10% neutral buffered formalin (NBF). The brains were extracted and incubated in NBF at 4°C for 24 h. Brains were then cryoprotected with increasing concentrations of sucrose (10, 20, and 30%) in PBS, allowing the brain to sink at each concentration (∼24 h). Following the final cryoprotection step, the brains were embedded in optimal cutting media (OCT) and rapidly frozen. The tissue was stored at −80°C until processing. For the juvenile tissue, 12-µm-thick sections were cut with a cryostat and mounted on charged slides. Slides were baked at 57°C for 20 min and then rinsed with PBS for 10 min to remove OCT. A hydrophobic barrier was drawn surrounding the tissue. Sections were incubated with blocking buffer (10% normal donkey serum, 0.5% Triton X-100) for 2 h at room temperature. Blocking buffer was removed, and sections were incubated with phospho-c-Fos (Ser32) primary antibody (1:1,000, D82C12, Cell Signaling Technology) at 4°C for 24 h. Slides were rinsed three times for 10 min in PBS prior to a 1 h incubation with secondary antibody, donkey anti-rabbit Alexa Fluor Plus 488 (1:500, A32790, Thermo Fisher Scientific). Slides were rinsed three times for 10 min in PBS and mounted with ProLong Gold Antifade Mountant with DAPI (Thermo Fisher Scientific). For the adult tissue, 40-µm-thick sections were cut with a cryostat and transferred to PBS on 12-well plates. Sections were incubated in blocking buffer for 2 h at room temperature prior to 48 h incubation in phospho-c-Fos (Ser32) primary antibody (1:500, D82C12, Cell Signaling Technology) at 4°C. Secondary antibody incubation (1:500, donkey anti-rabbit Alexa Fluor Plus 488) was for 2 h at room temperature prior to mounting. Images were acquired with a Leica Mica microscope at 20×. Analysis was performed with Imaris 10 (Oxford Instruments), quantifying the number of c-Fos–positive nuclei within a 500-µm-wide column through all layers of the barrel cortex.

### RNAscope

To measure 5-HT_1B_ receptor expression specifically in PV neurons of PND 13–14 rats, RNAscope in situ hybridization was performed in conjunction with immunohistochemistry on 12-µm-thick sections from the fixed-frozen tissue according to the manufacturer supplied protocol (ACD, MK 51-150). Following antigen retrieval (ACD Co-Detection Target Retrieval Solution, 5 min, 98–100°C), sections were incubated overnight at 4°C with anti-PV antibody (1:2500, PA1-933, Thermo Fisher Scientific). RNAscope hybridization was performed using probe 5-hydroxytryptamine receptor 1B (Htr1b, 420361, ACD) and fluorophore Opal 650 (1:1,000, Akoya Biosciences). There was a 30 min incubation with secondary antibody donkey anti-rabbit Alexa Fluor Plus 488 (1:250, A32790, Thermo Fisher Scientific) prior to mounting with ProLong Gold Antifade Mountant with DAPI (Thermo Fisher Scientific). Images of the barrel cortex were acquired with a Leica SP8 confocal microscope at 40×. Quantification of 5-HT_1B_ transcript puncta colocalized with PV immunohistochemistry labeling was performed using Imaris 10 (Oxford Instruments). Only cells labeled with PV immunohistochemistry were included in the analyses.

### RNA isolation and RT-qPCR

Tissue punches were collected from the barrel cortex of PND 12–14 rats bilaterally with a 15 gauge punch and stored at −80°C until processing. RNA was extracted and isolated using TRIzol reagent (Thermo Fisher Scientific) and the MicroElute total RNA kit (Omega Bio-tek) according to the manufacturer instructions. RNA concentrations were measured on a NanoDrop spectrophotometer, and 400 ng of cDNA was synthesized using an iScript cDNA synthesis kit (Bio-Rad Laboratories). mRNA expression was measured by RT-qPCR with Perfecta SYBR Green FastMix (Quanta) and a CFX384 system (Bio-Rad Laboratories) using the ΔΔCT method. GAPDH was used as the housekeeping gene. The primer sequences were as follows: 5-HT_1B_ receptor (Htr1b, NM_022225.3) forward 5′-AGAAGAAACTCATGGCCGCT and reverse 5′-GGGGAGCCAGCACACAATAA and GAPDH (Gapdh, NM_017008.4) forward 5′-TGGCCTCCAAGGAGTAAGAA and reverse 5′-TGTGAGGGAGATGCTCAGTG.

### Statistical analysis

Statistical analyses were conducted with GraphPad Prism 10. Statistical significance was set as *p* < 0.05. Parametric tests were used if the appropriate assumptions were met. Otherwise, nonparametric tests were used. The statistical tests used, the test statistic, and *p* values are listed in each figure legend. Error bars represent mean ± 95% confidence interval (CI) as noted in each figure legend. A nested *t* test was used for means comparisons to limit pseudoreplication. There were no differences between rats of different litters in the reported endpoints; thus they were combined for analysis. The source litter and sex of each experimental animal is listed in Table 1. The tissue for RT-qPCR experiments were collected across six litters per treatment group. In all experiments we adhere to accepted standards for rigorous study design and reporting to maximize the reproducibility and translational potential of our findings, as described in Landis et al. (2012) and in ARRIVE (Animal Research: Reporting In Vivo Experiments) Guidelines. We performed a power analysis to estimate the minimum sample size for each experiment using the G*Power software (Faul et al., 2007), with the desired alpha set to 0.05 and the desired power set to 0.80. The results reported for the c-Fos and 5-HT_1B_ RNAscope experiments reached statistical power at the group level but should be interpreted as preliminary as the sample sizes lacked sufficient power at the animal level. Additionally, sample sizes were not sufficiently powered to test for sex differences.

**Table 1.** Litter number and sex of individual experimental animals

	Vehicle	CPF
Figure 1*B*	4F, 14F, 13M	12F, 7M, 7M
Figure 2*B*, juvenile	4F, 5F, 13F, 4M	1F^a^, 2F, 11F, 2M
Figure 2*B*, adult	8F, 9F, 9F	6F, 10F, 10F
Figure 3*B*	4F, 5F, 13F, 3M, 4M	1F, 2F, 11F, 1M, 2M

M, male; F, female.

a Outlier excluded from analysis.

## Results

### Spontaneous neuronal activity

**Figure 1.**
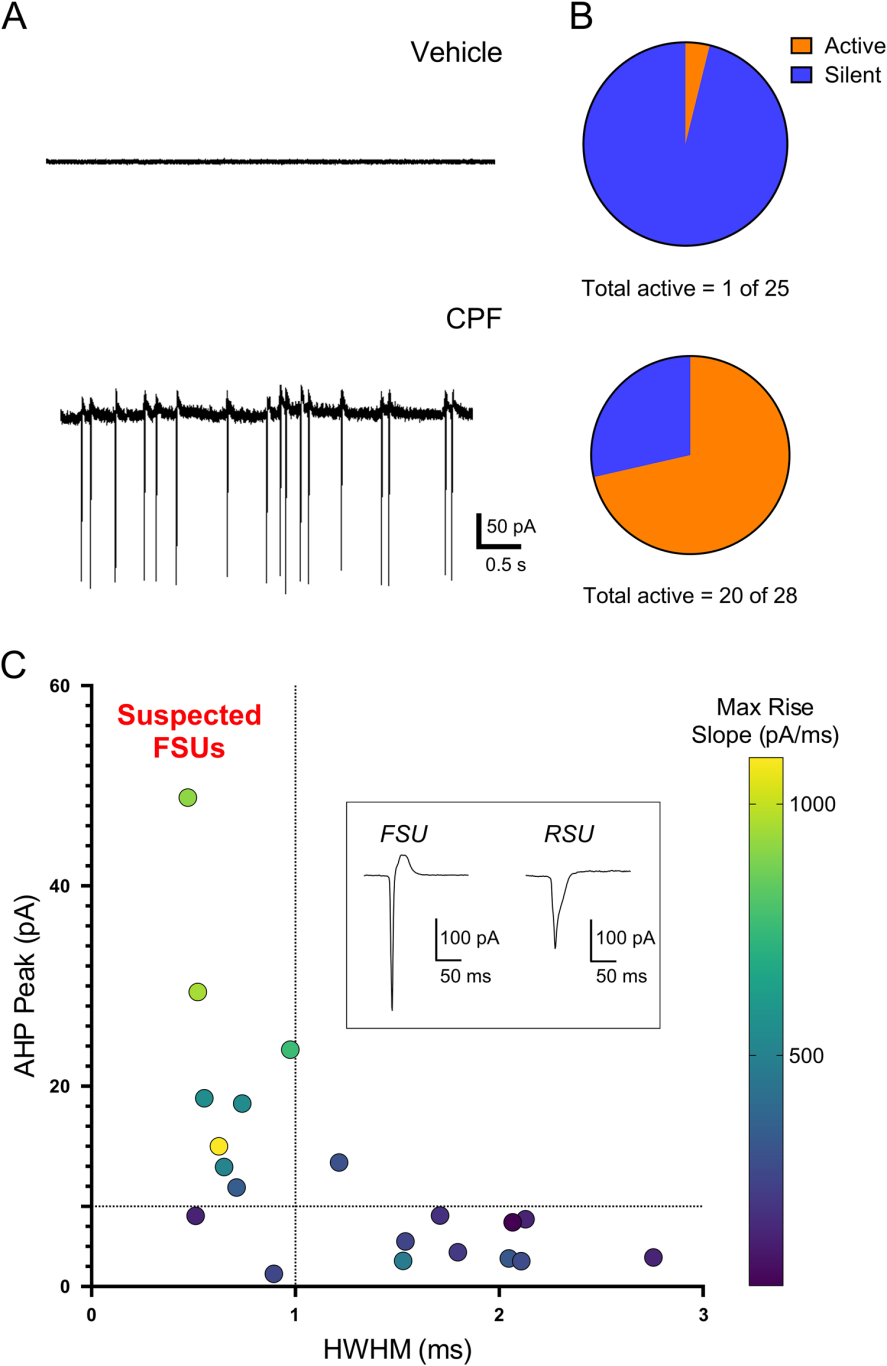
Gestational CPF exposure increases spontaneous activity of interneurons in the somatosensory (barrel) cortex. ***A***, Example cell-attached recording traces of suspected interneurons from vehicle- and CPF-exposed animals (PND 12–20). ***B***, A greater proportion of neurons had spontaneous activity (*p* < 0.0001, Fisher's exact test) in the treated (CPF) group [20 of 28 neurons from 3 animals (M, 10 of 12; M, 4 of 7; F, 6 of 9)] compared with vehicle [1 of 25 neurons from 3 animals (F: 0 of 4, F: 1 of 10, M: 0 of 11)]. ***C***, A subset of spontaneously active neurons (8 of 20) were identified as suspected FSUs based on their AHP peak being >8 pA and having an action potential half-width (HWHM) <1 ms. Inset shows example action potential traces from a FSU and a RSU.

Gestational CPF exposure results in a lasting increase in inhibitory inputs to pyramidal neurons (Koenig et al., 2025). To determine if this is driven by increased spontaneous activity of inhibitory neurons, we employed a cell-attached voltage–clamp technique in a brain slice preparation from juvenile (PND 12–20) rats. Interneurons were targeted based on their morphology in the superficial layers (Layers 2/3 and Layer 4) of the primary somatosensory (barrel) cortex. Example cell-attached traces from a vehicle and CPF-treated animal are seen in Figure 1*A*. Action potential currents can be seen in the representative trace from a CPF-exposed animal; these are absent in the neuron from the vehicle-treated animal. There was a greater proportion of neurons displaying spontaneous activity (Fig. 1*B*) in animals that had gestational CPF exposure (20 of 28, 71%) compared with neurons from vehicle-treated animals (1 of 25, 4%). These findings demonstrate that gestational CPF exposure results in a persistent increase in spontaneous neuronal activity of suspected inhibitory neurons.

We used previously established metrics to distinguish PV fast-spiking units (FSUs) from regular-spiking units (RSUs; Connors et al., 1982; McCormick et al., 1985; Murray and Keller, 2011). Figure 1*C* (inset) shows an example FSU with a characteristic short action potential duration and prominent afterhyperpolarization (AHP), absent from the RSU. Neurons with both an AHP peak >8 pA and a half-width at half-maximum (HWHM) <1 ms were designated as suspected FSUs. Of the 20 spontaneously active neurons in the CPF group, 8 were characterized as FSUs and 12 as RSUs (Fig. 1*C*). Those identified as RSUs could comprise pyramidal neurons or other classes of interneurons, such as somatostatin or serotonin receptor 5HT_3A_ expressing (Rudy et al., 2011).

### c-Fos expression

**Figure 2.**
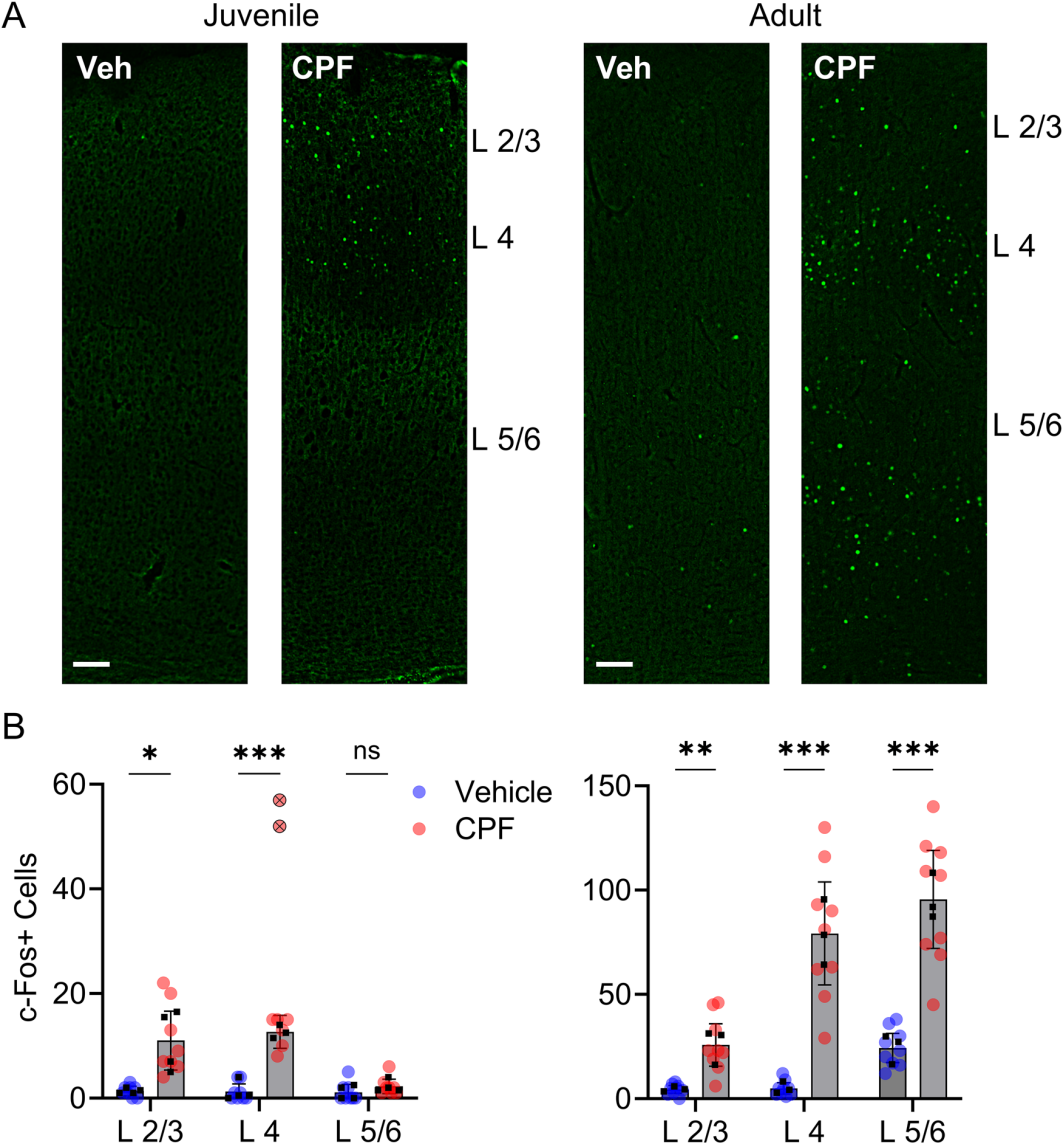
Gestational CPF exposure increases c-Fos expression in both juvenile and adult animals. ***A***, Representative immunohistochemistry images of the barrel cortex of juvenile (PND 12–13) and adult (>PND 90) animals showing c-Fos–positive nuclei. Scale bar, 100 µm. ***B***, The number of c-Fos–positive nuclei is higher in CPF-exposed juvenile animals [*n* = 8 sections from 4 animals (3 females, 1 male) per treatment] in Layers 2/3 (*t*_(6)_ = 3.3; *p* = 0.02) and Layer 4 (*t*_(5)_ = 9.0; *p* < 0.001) but not Layer 5/6 (*t*_(6)_ = 1.3; *p* = 0.25). In adult animals [*n* = 9 sections from 3 animals (3 females) per treatment], that number is higher in Layers 2/3 (*t*_(4)_ = 4.3; *p* = 0.01), Layer 4 (*t*_(4)_ = 6.9; *p* = 0.002), and Layer 5/6 (*t*_(4)_ = 6.7; *p* = 0.003, nested *t* test). There were no differences between the nested subgroups. The black squares represent the average number of c-Fos–positive nuclei for each individual animal. Outlier data points from the same animal excluded from analysis are marked with an X. Mean ± 95% CI (***B***).

As a second metric for increased neuronal activity, we evaluated increases in the expression of phosphorylated c-Fos, a product of an immediate early gene that serves as a reliable marker of neuronal activity and that is quickly upregulated following neuronal activation (Morgan et al., 1987; Dragunow and Faull, 1989). In both juvenile and adult vehicle-exposed offspring, labeling of c-Fos–positive nuclei was sparse across all layers (Fig. 2*A*,*B*). In contrast, in the CPF-exposed offspring, there was a marked increase in c-Fos labeling relative to vehicle-treated. In the juvenile age group, the numbers of c-Fos–positive nuclei were eightfold higher in Layers 2/3, 10-fold higher in Layer 4, but remained the same in layers 5/6 of the barrel cortex of offspring exposed to CPF compared with vehicle. In the adult age group, numbers of c-Fos–positive nuclei were 6-fold higher in Layers 2/3, 16-fold higher in Layer 4, and fourfold higher in Layers 5/6 of the barrel cortex of offspring exposed to CPF compared with vehicle (Fig. 2*B*). These data further support the hypothesis that CPF exposure results in persistent enhanced activation of cortical neurons.

### 5-HT_1B_ receptor expression

A potential mechanism driving both the increased spontaneous activity of neurons seen here and in previously reported enhancement of inhibitory synaptic transmission (Koenig et al., 2025) is presynaptic disinhibition of PV neurons. As stated above, there is a persistent elevation of binding to 5-HT_1A_ and 5-HT_2_ receptors in the brain of rats following gestational CPF exposure (Aldridge et al., 2003, 2004). While the 5-HT_1A_ receptor is both inhibitory and presynaptically expressed, it functions primarily as an autoreceptor on 5-HT neurons (Verge et al., 1985; Altieri et al., 2013). The 5-HT_1B_ receptor acts presynaptically to suppress transmitter release and is highly expressed on inhibitory neurons, potentially driving this disinhibition (Bramley et al., 2005; Egeland et al., 2011). Increased activity of 5-HT_1B_ receptors on inhibitory neurons could result in the disinhibition of interneurons that receive synaptic inputs from those inhibitory neurons. Lasting changes in the expression of 5-HT_1B_ receptors after prenatal CPF exposure have yet to be studied.

**Figure 3.**
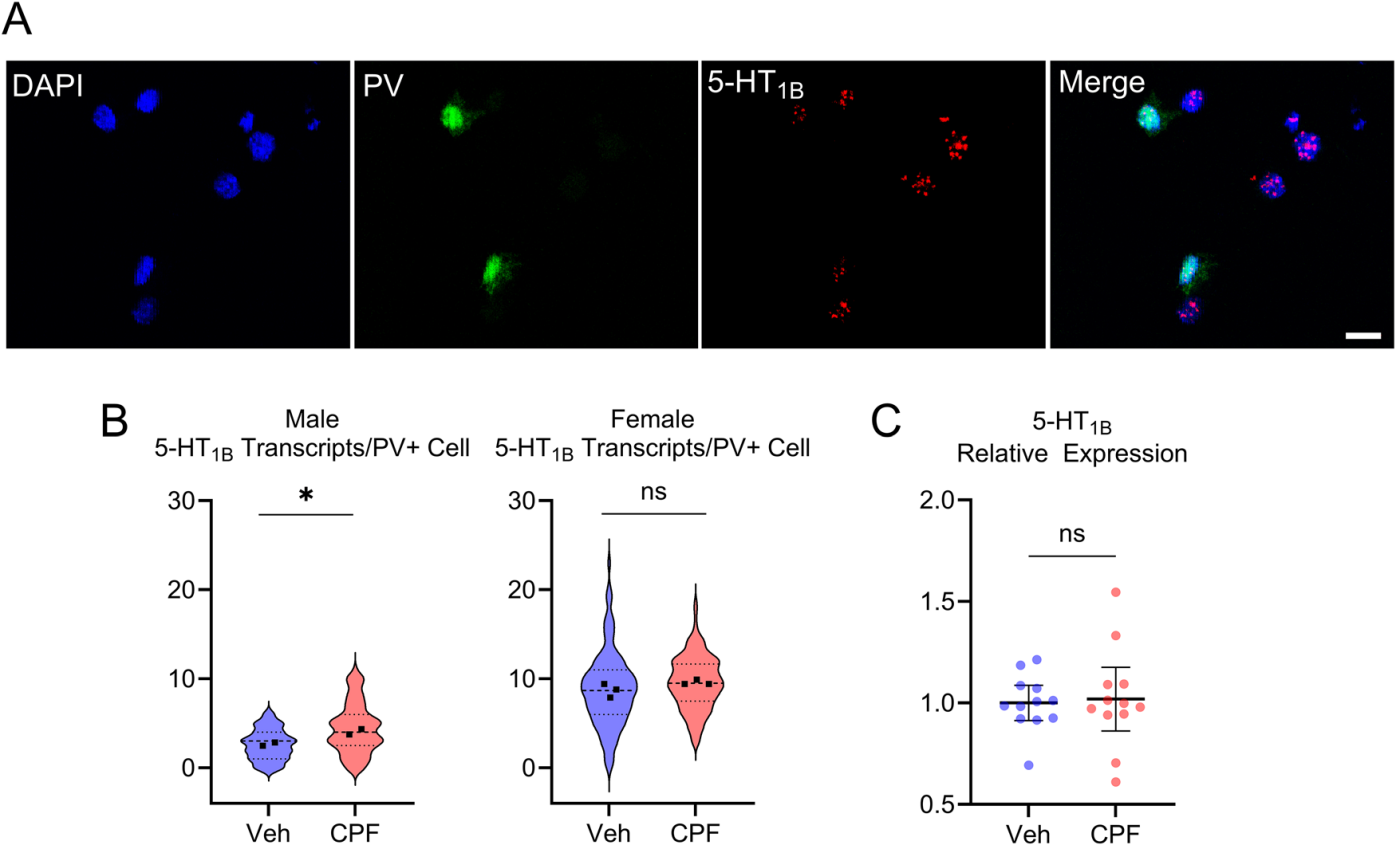
Gestational CPF exposure increases expression of the 5-HT_1B_ receptor in PV neurons. ***A***, Representative images showing immunohistochemistry labeling of PV interneurons and in situ hybridization RNAscope labeling of 5-HT_1B_ receptor transcripts in the barrel cortex. Scale bar, 10 µm. ***B***, Higher 5-HT_1B_ receptor expression following CPF exposure in males (*t*_(2)_ = 4.35; *p* = 0.049; vehicle *n* = 62 cells from 2 animals, CPF *n* = 116 cells from 2 animals) but not in females (*t*_(4)_ = 1.73; *p* = 0.16; vehicle *n* = 251 cells from 3 animals; CPF *n* = 315 cells from 3 animals, nested *t* test). There were no differences between the nested subgroups. The black squares represent the average number of transcripts per PV interneuron for each individual animal. ***C***, 5-HT_1B_ receptor expression in the barrel cortex measured by RT-qPCR did not demonstrate a difference (*t*_(17.2)_ = 0.23; *p* = 0.82; Welch's unpaired *t* test) between vehicle [(*n* = 12 animals (7 males, 5 females)] and CPF [*n* = 12 animals [9 males, 3 females)] groups. Violin plot with median and quartiles (***B***). Mean ± 95% CI (***C***).

Here, we employed RNAscope in situ hybridization to label 5-HT_1B_ transcripts, in conjunction with immunohistochemistry labeling of PV neurons in the barrel cortex. Figure 3*A* shows a representative image of immunohistochemistry-labeled PV neurons (green) colocalized with 5-HT_1B_ transcripts (red puncta). Each puncta corresponds to a single 5-HT_1B_ mRNA transcript (Wang et al., 2012). Quantification of puncta specifically colocalized with PV labeling (Fig. 3*B*) revealed 65% higher number of transcripts per cell in male offspring exposed to CPF compared with vehicle (veh, 2.6 ± 0.46; CPF, 4.3 ± 0.50) and 9% greater number of transcripts per cell in female offspring exposed to CPF compared with vehicle (veh, 8.7 ± 0.61; CPF, 9.5 ± 0.51). When comparing by sex, vehicle-exposed females had a 236% greater number of 5-HT_1B_ transcripts per PV neuron than vehicle-exposed males, and CPF-exposed females had a 122% greater number of transcripts per PV neurons than CPF-exposed males. Due to the limited number of animal replicates reported here (two males and three females per treatment group), these results should be interpreted as preliminary.

Measurement of total 5-HT_1B_ expression in the barrel cortex using RT-qPCR revealed no difference in the relative expression between vehicle (1.0 ± 0.07) and CPF exposed (1.0 ± 0.16) groups (Fig. 3*C*). The lack of difference seen in these results could be attributed to the nonspecific nature of the expression data. If changes in 5-HT_1B_ expression were limited to specific cell types, such as PV neurons, this global measurement could effectively washout any difference. Overall, these findings support the role of increased expression of the 5-HT_1B_ receptor, particularly in inhibitory PV neurons, in driving spontaneous neuronal activity following CPF exposure.

## Discussion

We hypothesized that gestational exposure to a subacute dose of CPF would result in lasting enhancement of spontaneous neuronal activity and an increased expression of the 5-HT_1B_ receptor, particularly in inhibitory neurons. Consistent with these predictions, there was a marked increase in the number of spontaneously active neurons in the barrel cortex, in conjunction with increased expression of c-Fos protein, a marker of neuronal activation. In situ hybridization labeling demonstrated an increase in 5-HT_1B_ expression in PV neurons following CPF exposure in males.

### Spontaneous neuronal activity

Altered inhibitory synaptic transmission following a subacute gestational exposure to CPF has been previously reported (Koenig et al., 2025). This presented as an increase in spontaneous inhibitory synaptic events but also as a reduction in presynaptic release probability of GABA onto Layer 2/3 excitatory pyramidal neurons. These phenomena could be explained by the disinhibition and increase in spontaneous firing of PV neurons, the primary inhibitory input to pyramidal neurons (Packer and Yuste, 2011; Yang et al., 2016). Here, we demonstrate this increase in spontaneous firing through cell-attached electrophysiology: 71% of the neurons recorded in CPF-exposed animals showed spontaneous firing, whereas only 4% of those in the control group fired spontaneously (Fig. 1*B*).

Only a subset of the spontaneously firing neurons (40%) were identified as PV fast-spiking neurons, based on their action potential metrics (Connors et al., 1982; McCormick et al., 1985). The remaining were identified as RSUs, and these may include excitatory neurons, as well as other classes of inhibitory cells, such as somatostatin-expressing neurons (Rudy et al., 2011). Somatostatin neurons, like their PV counterparts, provide inhibitory input to Layer 2/3 pyramidal neurons (Adesnik et al., 2012; Xu et al., 2013), and an increase in their spontaneous firing may contribute to the increase in inhibitory inputs reported previously (Koenig et al., 2025).

PV neurons reach anatomical and functional maturity only in late adolescence, and their maturation is critically sensitive to use-dependent changes in activity (Miller et al., 2011; Dehorter et al., 2015). Dysregulation of PV neuron activity is causally linked to a number of neuropsychiatric disorders, such as schizophrenia, autism spectrum disorder, substance use disorders, and depression (Ferguson and Gao, 2018; Selten et al., 2018; Filice et al., 2020; Nahar et al., 2021). Imbalance in excitatory/inhibitory synaptic transmission has also been implicated in these pathologies (Gao and Penzes, 2015; Ferranti et al., 2024). The hyperexcitability reported here could underly the persistent neurobehavioral detriments associated with early-life CPF exposure.

### c-Fos expression

As an additional metric to evaluate enhanced neuronal excitability, we measured c-Fos expression in the barrel cortex of juvenile and adult animals. In agreement with previous reports (Chu et al., 2013), there was only low c-Fos expression in vehicle-treated animals. The marked increase in c-Fos expression in CPF-exposed animals reported here appeared similar to animals who have undergone robust vibrissae stimulation (Filipkowski et al., 2000).

In juvenile animals that had gestational exposure to CPF, c-Fos labeling revealed neuronal activation in Layers 2/3 and 4, but not in deep Layer 5/6. This suggests enhanced activity related to thalamocortical activation in Layers 2/3 and 4, but not in the summation and output of Layers 5/6 (Keller, 1995; Laaris et al., 2000). During the second week of postnatal development, the age of juvenile animals in the present study, Layer 2/3 neurons, receive more excitatory drive, compared with Layer 5 neurons (Kroon et al., 2019). Furthermore, synapses onto Layer 2/3 neurons develop earlier than synapses from Layer 3 to Layer 5 (Clement et al., 2013). Both phenomena could account for the absence of c-Fos labeling in these deep layers of juvenile animals. The robust level of c-Fos expression in adult animals, >90 d following the brief gestational exposure, also supports the persistent nature of these CPF-induced neurophysiological alterations.

The cell types of the c-Fos–positive cells were not identified in this experiment. Previous reports have shown that both inhibitory and excitatory cells increase c-Fos expression when exposed to a novel environment (Staiger et al., 2002). An increase in c-Fos expression in PV or other inhibitory neurons is consistent with previously reported electrophysiology results, showing enhanced inhibitory synaptic activity in the barrel cortex after gestational CPF exposure (Koenig et al., 2025). However, it is also possible that amplified inhibitory inputs result in paradoxical hyperexcitation of excitatory, pyramidal neurons, as a result of postinhibitory rebound spiking (Morishima et al., 2017; Moore et al., 2018). As pyramidal and PV neurons form reciprocal connections, this could lead to abnormal response properties of excitatory cortical neurons and affect cortical oscillations that facilitate cognition and attention (Sohal et al., 2009; Kim et al., 2016; Tan et al., 2019).

Hypersensitivity to sensory stimulation is a common phenotype and hallmark in individuals with autism spectrum disorder (Markram and Markram, 2010). Animal models of fragile X syndrome and valproic acid-induced autism phenotypes have consistently demonstrated persistent hyperexcitability in the somatosensory cortex (Deng and Lei, 2008; Markram and Markram, 2010). The enhanced neuronal spontaneous activity and increased basal c-Fos expression reported here both support this phenomenon and potentially underly the increased incidence of these neurobehavioral detriments seen in populations exposed gestationally to CPF. Additional experiments will be needed to identify a causal relationship between these phenomena.

### 5-HT_1B_ receptor expression

We have previously reported a reduction in the presynaptic release probability of GABA in CPF-exposed animals. We hypothesized this is driven by increased expression of the inhibitory receptor 5-HT_1B_, specifically on PV neurons. As the primary inhibitory input onto PV neurons is other PV neurons (Kubota et al., 2016; Yang et al., 2016), presynaptic disinhibition could lead to their overall increased activity described above. Our in situ RNAscope results support this, demonstrating increased 5-HT_1B_ expression in PV neurons of our CPF-exposed male animals.

Previous reports have demonstrated altered activity of receptor subtypes 5-HT_1A_ and 5-HT_2_, in addition to the 5-HT transporter (SERT), following gestational CPF exposure (Aldridge et al., 2003, 2004). The inhibitory 5-HT_1A_ receptor primarily functions as an autoreceptor on 5-HT neurons (Verge et al., 1985; Altieri et al., 2013); we did not focus on this receptor in the current study. In the present study, we focused on the receptor subtype 5-HT_1B_. This receptor is preferentially expressed on presynaptic axon terminals, and its activation inhibits GABA release (Boschert et al., 1994; Matsuoka et al., 2004; Bramley et al., 2005), aligning with our proposed model of disinhibition of PV neurons. The 5-HT_1B_ receptor also plays a critical role in the proper patterning of the rodent barrel cortex (Cases et al., 1996; Rebsam et al., 2002). This altered 5-HT_1B_ expression induced by CPF exposure could be a mechanism driving the sensory processing disorders associated with early OP exposure (Cartier et al., 2018; Silver et al., 2018). It also supports prior findings of alterations to barrel field patterning using this same CPF exposure model (Koenig et al., 2025). Whereas previous studies on the lasting effects of gestational CPF exposure (Aldridge et al., 2003, 2004) only reported changes in global 5-HT receptor activity in particular brain regions, the PV neuron-specific alterations reported here provide mechanistic framework to allow for further interrogation of the role that 5-HT_1B_ receptors might have on the developmental neurotoxicity of CPF.

Basal 5-HT_1B_ expression was markedly higher in females compared with males. This is consistent with previous reports of higher basal levels of 5-HT synaptic proteins (5-HT_1A_ and 5-HT_2_) in females compared with males (Slotkin and Seidler, 2005). It is also consistent with findings that female knock-out 5-HT_1B_ mice exhibit more pronounced stress-induced depressive behaviors compared with males (Jones and Lucki, 2005). Here, we report that prenatal CPF exposure resulted in upregulation of 5-HT_1B_ RNA expression in PV neurons only in males. This may reflect sex differences in the consequences of CPF exposure. Previous reports of sex differences in 5-HT receptor expression in response to perinatal CPF exposure have been mixed, depending on the targeted brain region (Aldridge et al., 2004; Slotkin and Seidler, 2005). It is also possible that our data reflect a “ceiling effect” resulting in the much higher 5-HT_1B_ expression levels in normal females or the larger variance in these levels in females (Fig. 3).

We recognize that some of the sample sizes in this study are small at the animal level. While we attempted to control for pseudoreplication using a hierarchical analysis (nested *t* test) where possible, these findings should be considered preliminary. The limited number of animals also precluded identifying sex-specific effects of CPF exposure.

### Conclusion

The present study furthers our understanding of the underlying neurophysiological effects of gestational exposure to CPF. The higher spontaneous neuronal activity and increased expression of 5-HT_1B_ receptor in PV neurons of male animals offer potential mechanisms driving the persistent changes in synaptic properties and plasticity previously described (Koenig et al., 2025) and the persistent neurobehavioral disorders associated with this exposure. Whether these effects are a direct consequence of CPF exposure and whether they reflect causal mechanisms underlying these detriments remains to be confirmed.

## Synthesis

Reviewing Editor: Jibran Khokhar, Western University Department of Anatomy and Cell Biology Decisions are customarily a result of the Reviewing Editor and the peer reviewers coming together and discussing their recommendations until a consensus is reached. When revisions are invited, a fact-based synthesis statement explaining their decision and outlining what is needed to prepare a revision will be listed below. The following reviewer(s) agreed to reveal their identity: NONE.

The manuscript investigates the neurodevelopmental consequences of prenatal exposure to chlorpyrifos (CPF), focusing on increased activity in fast-spiking interneurons (FSIs), elevated c-Fos expression, and enhanced 5-HT1B receptor transcript levels in PV-positive cells in the somatosensory cortex. The study addresses an important knowledge gap and attempts to provide mechanistic insights into how gestational CPF exposure leads to long-term changes in cortical function. However, reviewers identified a central interpretive gap: the evidence linking changes in 5-HT1B receptor expression in PV cells to the observed cortical hyperactivity remains correlational. In addition, concerns were raised regarding the rationale for receptor selection, environmental relevance of the CPF dose, statistical reporting, and clarity of data presentation. Overall, the study presents promising data but requires revision and clarification to support its conclusions more convincingly.

⸻

Major Concern

The manuscript proposes that increased expression of the 5-HT1B receptor in PV-positive interneurons mediates the observed increase in cortical excitability, as indexed by spontaneous firing and c-Fos expression. However, the presented data are correlational and do not demonstrate a causal link between receptor upregulation and altered neuronal activity. The study would benefit from additional experiments or analyses to strengthen this connection. For instance, demonstrating that the c-Fos+ cells are PV neurons or that these neurons also express elevated 5-HT1B receptors would provide more direct support. Alternatively, targeted pharmacological or genetic manipulation of 5-HT1B receptors in PV cells could help establish causal involvement. Clarifying whether the authors view these molecular and electrophysiological changes as independent consequences of CPF or as parts of a mechanistically linked pathway is also essential for interpreting the results.

⸻

Minor Concerns

Introduction

The justification for focusing on the 5-HT1B receptor over other inhibitory serotonin receptors is insufficient. While the manuscript implies that 5-HT1B is relevant, the basis for excluding other candidate receptors such as 5-HT1A and 5-HT2A is not clearly articulated. These other receptors are known to modulate interneuron physiology and could be equally relevant. To improve clarity, the introduction should include a concise rationale for focusing on this receptor subtype, supported by references to prior studies that demonstrate its specific role in regulating FSIs or PV neuron excitability. Additionally, since the study is conducted in the barrel cortex, a region with well-documented developmental serotonergic modulation, the absence of any mention of the serotonin transporter (SERT) or its interaction with 5-HT1B receptors is a notable omission. This context should be addressed either in the introduction or discussion.

The framing of CPF exposure also requires revision. The current language suggests that the CPF dose used in the study does not significantly inhibit acetylcholinesterase, which is inconsistent with prior data. Published studies using this dose have shown substantial and sustained inhibition of brain AChE. The introduction should reflect this and clarify that while lower exposures may not inhibit AChE, the dose used here is above that threshold.

⸻

Materials and Methods

Concerns were raised about the environmental relevance of the CPF dose. While the selected dose has precedent in the literature, the extent to which it reflects current human environmental exposures, particularly in North America, is unclear. The authors should either provide updated citations supporting the relevance of this exposure level or acknowledge that the chosen dose models high or accidental exposures and is primarily used to probe underlying mechanisms.

The use of DMSO as part of the vehicle is also problematic. DMSO has known biological activity, including potential toxicity and the ability to enhance cellular uptake of xenobiotics. Its inclusion in the injection mixture introduces the possibility of off-target effects or altered pharmacokinetics of CPF. The authors should justify this vehicle choice, reference toxicity thresholds where applicable, and explain why a non-DMSO solvent like oil alone was not used.

The methods also lack detail in some areas. The process for culling litters should be described more precisely-specifically, whether pups were selected randomly or based on size. The term "antigen retrieval" is used in the RNAscope section without adequate explanation and should be clarified. Furthermore, the age of animals used for slice electrophysiology is not clearly stated, which is important for aligning this data with c-Fos analyses performed at different timepoints.

Statistical methods are described in general terms but do not include specifics about how assumptions were tested, whether transformations were considered for non-normally distributed data, or how model effects were handled. The methods should specify what statistical models were used, whether parametric or non-parametric tests were chosen, and how interactions between variables like sex and treatment were analyzed. Additional information about multiple comparison corrections, if any, would also be helpful.

⸻

Results

The results section lacks sufficient statistical detail. Differences between treatment groups are described without reporting test statistics, degrees of freedom, or p-values. This omission makes it difficult to evaluate the robustness of the findings. Additionally, the presentation of sex differences is inconsistent with the statistical outcomes. Although differences are described between males and females, the manuscript acknowledges that there was no significant sex-by-treatment interaction. Unless analyses are performed separately by sex and show statistically significant effects, these comparisons should not be emphasized in the text or figures.

The visual presentation of data also needs improvement. Figure 3B, which displays 5-HT1B transcript counts per PV+ cell, uses a violin plot that does not effectively highlight group differences. Reviewers found this format difficult to interpret and recommended using a bar plot or other more intuitive visualization. Moreover, the image shown in Figure 3A does not convincingly illustrate elevated receptor expression in PV neurons. The signal appears stronger in non-PV cells, which raises concerns about the validity of the quantification. A clearer example with robust co-localization of signal in PV cells would better support the interpretation.

Reviewers also questioned the unit of analysis in the statistical evaluation of transcript counts. Using individual cells as independent observations, particularly when only a few animals per group are included, can artificially inflate degrees of freedom and lead to misleading conclusions. The analysis should account for hierarchical data structure and use animals, not cells, as the statistical unit when appropriate.

⸻

Discussion

The discussion is generally well structured and clearly written but is weakened by the lack of robust statistical support in the results. Without clearer presentation of data and analysis, some of the conclusions appear overstated. For example, the suggestion that the findings represent potential causal mechanisms is valid in principle, but the manuscript does not yet provide sufficient evidence to support mechanistic links among the observed outcomes. The discussion should clarify whether the proposed mechanisms are speculative or directly supported by the data.

Interpretations related to sex differences require refinement. If no significant interaction between sex and treatment was found, then conclusions about sex-specific effects should be avoided or appropriately qualified. Likewise, numerical differences in transcript counts between sexes do not justify mechanistic claims unless supported by statistically valid comparisons.

Finally, the manuscript should more explicitly address whether the observed effects are thought to be connected in a causal sequence or represent independent consequences of CPF exposure. This distinction is important for future work and should be clearly articulated in the concluding remarks.

## Author Response

Dear Editor, Thank you for the invitation to resubmit our manuscript and the opportunity to address the reviewers' comments. We thank the reviewers for their thoughtful comments which we believe improve the quality of our manuscript. We address all comments below and have uploaded a revised version of the manuscript with changes highlighted in yellow.

Major Concern The manuscript proposes that increased expression of the 5-HT1B receptor in PV-positive interneurons mediates the observed increase in cortical excitability, as indexed by spontaneous firing and c-Fos expression. However, the presented data are correlational and do not demonstrate a causal link between receptor upregulation and altered neuronal activity. The study would benefit from additional experiments or analyses to strengthen this connection. For instance, demonstrating that the c-Fos+ cells are PV neurons or that these neurons also express elevated 5-HT1B receptors would provide more direct support. Alternatively, targeted pharmacological or genetic manipulation of 5-HT1B receptors in PV cells could help establish causal involvement. Clarifying whether the authors view these molecular and electrophysiological changes as independent consequences of CPF or as parts of a mechanistically linked pathway is also essential for interpreting the results.

The reviewers are correct in that the data we present are correlational and do not represent causal links between chlorpyrifos exposure, changes in 5-HT receptor expression, and neuronal activity patterns. Our aim, at this early stage of this research project, was not to test for causality. Rather, our goal was to determine the lasting effects of this exposure on cortical synaptic functions. Testing causality is an important question that will be addressed in our future studies. These studies will include the excellent suggestions by the reviewers to identify the neuronal classes that express increased c-Fos, and to determine if the elevated 5-HT receptor expression occurs specifically in parvalbumin neurons. We have modified relevant statements throughout the text to emphasize that our results are correlational.

Minor Concerns Introduction The justification for focusing on the 5-HT1B receptor over other inhibitory serotonin receptors is insufficient. While the manuscript implies that 5-HT1B is relevant, the basis for excluding other candidate receptors such as 5-HT1A and 5-HT2A is not clearly articulated. These other receptors are known to modulate interneuron physiology and could be equally relevant. To improve clarity, the introduction should include a concise rationale for focusing on this receptor subtype, supported by references to prior studies that demonstrate its specific role in regulating FSIs or PV neuron excitability. Additionally, since the study is conducted in the barrel cortex, a region with well-documented developmental serotonergic modulation, the absence of any mention of the serotonin transporter (SERT) or its interaction with 5-HT1B receptors is a notable omission. This context should be addressed either in the introduction or discussion.

The reviewers are correct in raising the potential importance of other candidate 5-HT receptors. We have added to the Discussion additional rationale for our focus on 5-HT1B in the current study: "Previous reports have demonstrated altered activity of receptor subtypes 5-HT1A and 5-HT2, in addition to the 5-HT transporter (SERT), following gestational CPF exposure (Aldridge et al., 2004, 2003). In the present study we focused on the receptor subtype 5-HT1B. This receptor is preferentially expressed on presynaptic axon terminals, and its activation inhibits GABA release (Boschert et al., 1994; Bramley et al., 2005; Matsuoka et al., 2004), aligning with our proposed model of disinhibition of PV neurons." The framing of CPF exposure also requires revision. The current language suggests that the CPF dose used in the study does not significantly inhibit acetylcholinesterase, which is inconsistent with prior data. Published studies using this dose have shown substantial and sustained inhibition of brain AChE. The introduction should reflect this and clarify that while lower exposures may not inhibit AChE, the dose used here is above that threshold.

The reviewers are correct that the selected dose significantly inhibits brain AChE activity in offspring. We have added text to the Material and Methods section to emphasize that this inhibition is transient, and that at the time points examined in our paper AChE activity returns to normal levels: "This exposure paradigm significantly inhibits brain AChE in offspring of treated dams. However, AChE enzymatic activity returns to control levels by PND 12 (Koenig et al., 2025), a period preceding the endpoints presented here." Materials and Methods Concerns were raised about the environmental relevance of the CPF dose. While the selected dose has precedent in the literature, the extent to which it reflects current human environmental exposures, particularly in North America, is unclear. The authors should either provide updated citations supporting the relevance of this exposure level or acknowledge that the chosen dose models high or accidental exposures and is primarily used to probe underlying mechanisms.

The reviewers raise an important concern on the relevance of the selected exposure dose of CPF. We have added text to the Material and Methods section clarifying this point: "The CPF exposure dose (5 mg/kg) was selected to probe mechanisms driving the persistent neurodevelopmental alterations associated with sub-acute, non-symptomatic, CPF exposure. A similar dose was used in a series of studies by Slotkin and collaborators (Aldridge et al., 2004, 2003; Slotkin and Seidler, 2007). ... We recognize that this dose does not necessarily reflect levels of environmental exposure in the human population." The use of DMSO as part of the vehicle is also problematic. DMSO has known biological activity, including potential toxicity and the ability to enhance cellular uptake of xenobiotics. Its inclusion in the injection mixture introduces the possibility of off-target effects or altered pharmacokinetics of CPF. The authors should justify this vehicle choice, reference toxicity thresholds where applicable, and explain why a non-DMSO solvent like oil alone was not used.

The reviewers are correct in that DMSO can have biological activity and potential toxicity. We have added text to the Materials and Methods section explaining the use of DMSO in this study: "Previous studies have routinely used DMSO (1.0 mL/kg) as the vehicle for CPF administration in rat dams (Aldridge et al., 2005; Garcia et al., 2003; Levin et al., 2001). The dose of DMSO administered here (0.25 mL/kg) was reduced to limit any potential off-target toxicity (Galvao et al., 2014). The use of an identical dose of DMSO in control animals further mitigates this concern. In addition, the dose of DMSO administered in the current study (0.25 mL/kg) is well below the highest no-observable-adverse-effect level (NOAEL) previously reported in rats (Gad et al., 2006)." The methods also lack detail in some areas. The process for culling litters should be described more precisely-specifically, whether pups were selected randomly or based on size. The term "antigen retrieval" is used in the RNAscope section without adequate explanation and should be clarified. Furthermore, the age of animals used for slice electrophysiology is not clearly stated, which is important for aligning this data with c-Fos analyses performed at different timepoints.

We have added text in the Materials and Methods section and the legend of Figure 1 clarifying these points: "Litters were culled to a maximum size of 10 by PND 3 through random pup selection." "Following antigen retrieval (ACD Co-Detection Target Retrieval Solution, 5 min, 98-100ºC)..." "As previously described (Alipio et al., 2021), animals (PND 12-20) were deeply anesthetized..." "Figure 1. Gestational chlorpyrifos exposure increases spontaneous activity of interneurons in the somatosensory (barrel) cortex. A. Example cell-attached recording traces of suspected interneurons from vehicle and CPF exposed animals (PND 12-20)." Statistical methods are described in general terms but do not include specifics about how assumptions were tested, whether transformations were considered for non-normally distributed data, or how model effects were handled. The methods should specify what statistical models were used, whether parametric or non-parametric tests were chosen, and how interactions between variables like sex and treatment were analyzed. Additional information about multiple comparison corrections, if any, would also be helpful.

Results The results section lacks sufficient statistical detail. Differences between treatment groups are described without reporting test statistics, degrees of freedom, or p-values. This omission makes it difficult to evaluate the robustness of the findings.

To address this and the above point, we included the statistical test information in each figure legend. We did not include these in the main text to avoid clutter and for ease of reading. We have added text to the Materials and Methods section to clarify the location of this information: "The statistical tests used, the test statistic, and p values are listed in each figure legend." Additionally, the presentation of sex differences is inconsistent with the statistical outcomes. Although differences are described between males and females, the manuscript acknowledges that there was no significant sex-by-treatment interaction. Unless analyses are performed separately by sex and show statistically significant effects, these comparisons should not be emphasized in the text or figures.

The reviewers raise an important concern on the presentation of sex differences. As suggested, we have modified the analyses and now present analyses separated by sex, using a nested t-test. We have modified text in the Discussion concerning these data: "Basal 5-HT1B expression was markedly higher in females, compared to males. This is consistent with previous reports of higher basal levels of 5-HT synaptic proteins (5-HT1A and 5-HT2) in females, compared to males (Slotkin and Seidler, 2005). It is also consistent with findings that female knockout 5-HT1B mice exhibit more pronounced stress-induced depressive behaviors, compared to males (Jones and Lucki, 2005). Here, we report that prenatal CPF exposure resulted in upregulation of 5-HT1B RNA expression in PV neurons only in males. This may reflect sex differences in the consequences of CPF exposure. Previous reports of sex differences in 5-HT receptor expression in response to perinatal CPF exposure have been mixed, depending on the targeted brain region (Aldridge et al., 2004; Slotkin and Seidler, 2005). It is also possible that our data reflect a 'ceiling effect' resulting in the much higher 5-HT1B expression levels in normal females, or the larger variance in these levels in females (Fig. 3)." The visual presentation of data also needs improvement. Figure 3B, which displays 5-HT1B transcript counts per PV+ cell, uses a violin plot that does not effectively highlight group differences. Reviewers found this format difficult to interpret and recommended using a bar plot or other more intuitive visualization.

Having considered and experimented with other data visualization methods, including box and bar plots, we believe the violin plot remains the best choice for this particular data set. Of the available options, the violin plot most clearly displays the shape of distribution (Hintze and Nelson, 1998).

Moreover, the image shown in Figure 3A does not convincingly illustrate elevated receptor expression in PV neurons. The signal appears stronger in non-PV cells, which raises concerns about the validity of the quantification. A clearer example with robust co-localization of signal in PV cells would better support the interpretation.

We failed to make clear that the analysis of 5-HT1B transcript counts was limited to cells that co-expressed parvalbumin. Non-PV cells were excluded from all analyses. Additional text was added to the Materials and Methods section clarifying this point. "Only cells labeled with PV immunohistochemistry were included in the analyses." Reviewers also questioned the unit of analysis in the statistical evaluation of transcript counts. Using individual cells as independent observations, particularly when only a few animals per group are included, can artificially inflate degrees of freedom and lead to misleading conclusions. The analysis should account for hierarchical data structure and use animals, not cells, as the statistical unit when appropriate.

The reviewers raise an important concern. To address this, we have modified the analysis of 5-HT1B transcript counts utilizing a nested t-test, with transcript count per cell nested within individual animals in each treatment group.

Discussion The discussion is generally well structured and clearly written but is weakened by the lack of robust statistical support in the results. Without clearer presentation of data and analysis, some of the conclusions appear overstated. For example, the suggestion that the findings represent potential causal mechanisms is valid in principle, but the manuscript does not yet provide sufficient evidence to support mechanistic links among the observed outcomes. The discussion should clarify whether the proposed mechanisms are speculative or directly supported by the data.

Interpretations related to sex differences require refinement. If no significant interaction between sex and treatment was found, then conclusions about sex-specific effects should be avoided or appropriately qualified. Likewise, numerical differences in transcript counts between sexes do not justify mechanistic claims unless supported by statistically valid comparisons.

Finally, the manuscript should more explicitly address whether the observed effects are thought to be connected in a causal sequence or represent independent consequences of CPF exposure. This distinction is important for future work and should be clearly articulated in the concluding remarks.

The reviewers are correct in the need to clarify the claims of potential causal mechanisms. For the above three points, we have modified the Discussion, qualifying all mechanistic claims and stating the need for future work to more fully support the identification of causal mechanisms induced by gestational CPF exposure: "The present study furthers our understanding of the underlying neurophysiological effects of gestational exposure to CPF. The higher spontaneous neuronal activity and increased expression of 5-HT1B receptor in PV neurons of male animals offer potential mechanisms driving the persistent changes in synaptic properties and plasticity previously described (Koenig et al., 2025), and the persistent neurobehavioral disorders associated with this exposure. Whether these effects are a direct consequence of CPF exposure, and whether they reflect causal mechanisms underlying these detriments, remains to be confirmed." - References Hintze, J.L., Nelson, R.D., 1998. Violin Plots: A Box Plot-Density Trace Synergism. The American Statistician 52, 181-184. https://doi.org/10.1080/00031305.1998.10480559
